# Mothers’ experiences living with diastasis recti abdominis – an interview study

**DOI:** 10.1186/s12905-024-03131-x

**Published:** 2024-05-17

**Authors:** Viktoria Marander, Målfrid Råheim, Inger Haukenes, Nina-Margrethe Theodorsen

**Affiliations:** 1 Department of Global Public Health and Primary Care, University of Bergen, Alrek helseklynge, blokk D, Årstadveien 17, Bergen, 5009 Norway; 2 Research Unit for General Practice, NORCE - Norwegian Research Centre, Bergen, Norway

**Keywords:** Provider-patient encounter, Self-image, Postpartum, Diastasis recti abdominis, Inter-rectus distance, Social media

## Abstract

**Background:**

Diastasis recti abdominis (DRA) is a common postpartum condition. Knowledge is scarce on how mothers perceive living with DRA. The interaction between healthcare providers and patients plays a significant role in shaping the healthcare service experience. Women suffering from typical women’s diseases tend to experience not being taken seriously or listened to when seeking healthcare. The aim of this study was to explore mothers’ experiences living with DRA.

**Methods:**

Semi-structured individual interviews were conducted with six Norwegian mothers, age 32–41, presenting with a clinically significant DRA. Topics discussed were how the condition is experienced, how it affects different aspects of day-to-day life and experiences with healthcare services. The data was analyzed using systematic text condensation.

**Results:**

DRA had an impact on everyday life among the mothers included in this study. Three major themes emerged: (I) The path to obtaining knowledge and understanding of DRA, (II) DRA - intertwined with health issues and physical limitations and (III) A changed belly – on self-image & social interactions. The mothers experienced uncertainties and frustration when trying to learn about DRA. The limited knowledge of the condition made it hard to differentiate if the experienced symptoms were caused by presence of DRA or from other health issues. Several mothers felt misunderstood.

**Conclusion:**

DRA is a multifaceted condition affecting many aspects of day-to-day life in various dimensions, like physical, emotional, and social. This study contributes to a wider understanding of living with DRA, which might guide healthcare professionals in providing support for mothers with this condition.

**Supplementary Information:**

The online version contains supplementary material available at 10.1186/s12905-024-03131-x.

## Background

Diastasis recti abdominis (DRA) is a common condition affecting mothers both pre- and postnatally. Most women experience DRA during the last trimester of pregnancy, and while DRA decreases during the postpartum period, the prevalence may still be 80% or more right after birth, 39–45% 6 months postpartum, and 33% 12 months postpartum [1–4]. Yet, the real prevalence rate is unknown due to various cut-off values, and different use of measurement methods and locations [5]. DRA occurs when the distance between the two muscle bulks of the rectus abdominis muscle is increased. This distance is called the inter-recti distance (IRD, and an increased IRD) may be due to a long-lasting increased intra-abdominal pressure, as with pregnancy, which causes a stretched and weakened linea alba [1, 6].

Research suggest that DRA is associated with pelvic floor prolapse, low back and pelvic area pain as well as lumbo-pelvic and pelvic floor dysfunction, including incontinence [7–9]. However, other studies have not found such correlations to DRA [3, 4, 10, 11]. Nevertheless, there seem to be an agreement that mothers with DRA have a higher prevalence of abdominal discomfort, pain, and decreased abdominal muscle strength than mothers without DRA [4, 7, 8, 11, 12]. Furthermore, there is scarce knowledge on how DRA is experienced among mothers, studies show that presence of DRA may reduce quality of life and amplify body dissatisfaction [2, 4, 8, 12].

There is a lack of international guidelines on how to prevent and treat DRA in the clinical setting. Carlstedt et al. [13] have only recently provided standardized recommendations for diagnosis and treatment of DRA in a Swedish setting. Thus, having no consensus on how to best approach DRA has led healthcare workers to treat mothers with DRA based on beliefs, attitudes, experiences, and information obtained online [14]. Exercising during pregnancy and the postpartum period is suggested to reduce the IRD [15]. Considering the high prevalence of DRA postpartum, the lack of clinical guidelines for treatment and limited knowledge on how DRA is perceived, healthcare professionals struggle to provide evidence-based care [14]. Eriksson Crommert et al. [16] suggest that the lack of guidelines may lead healthcare professionals, who provide care for women with DRA, to diminish the problems presented and/or seek support in undocumented treatment regimens. This may in turn lead mothers with DRA seeking information not supported by evidence or avoiding to seek help despite having persistent problems [16].

Health literacy is the ability to obtain, understand, process, and apply health information to promote and maintain good health, which further may improve people’s access and capacity to utilize health information [17, 18]. The conceptual model of health literacy may add a more comprehensive understanding to how mothers with DRA understand and seek help for the condition [18]. Moreover empowerment relies on a fundamental understanding of health literacy [17, 18]. Empowerment, in health promotion is the process where one may enhance health by achieving greater control over the decisions and actions affecting one’s life and health [17].

Patient satisfaction is influenced by how the provider-patient encounter is perceived [19]. Upmark et al. [20] suggests that women, to a greater extent than men, perceive being met with disrespectful behavior like nonchalance, disbelief or feeling not listened to in the encounter with healthcare providers. More knowledge of mothers’ experiences living with DRA will add an educated backdrop to the provider-patient encounter, and contribute to better starting point when exploring mothers concerns and providing support. Such knowledge may also help mothers with DRA live better lives. By narrowing the knowledge gap regarding how DRA is experienced among mothers this study seeks to contribute to a wider understanding concerning women’s health.

The aim of this study was to explore mothers’ experiences living with DRA.

## Methods

### Design and participants

This is a qualitative interview study with the intent to explore meaning across cases. This study is part of an ongoing project, *Prevention and treatment of Diastasis Rectus Abdominis during pregnancy and the postpartum period* (Regional Committee for Medical Ethics in Norway approval 76,296), where women were recruited through local hospitals, health centers, clinics and social media. The inclusion criteria were a clinically significant DRA [6, 21], with an IRD of 28 mm or more. Mothers who agreed to be contacted for further studies, were asked to participate in the present study. Three mothers were recruited from the main study. Another three mothers were recruited when they contacted the main supervisor of the project asking to contribute with their experiences.

Purposive sampling and the concept of information power was applied to achieve variation in the sample and data collected [22, 23]. A larger sample size is required to strengthen information power if the aim of a study is broad [23]. The interviews brought forth a variety of experiences and meanings despite the small sample size. Moreover the conceptual model of health literacy supported and elaborated the discussion concerning how the mothers understand and seek help for DRA [23]. The displayed diversity, various ages and various severity of DRA, within the group was considered to strengthen information power. Although the author who conducted the interviews have counseling experience, pilot interviews were undertaken to practice establishing trust and understanding [23]. This was considered contributing to improvement in quality of dialogue.

The mothers were 32–41 years old, had 2–3 children and presented an IRD between 2,8 and 11 cm. Two mothers had previously undergone caesarean section. All were 3–12 months postpartum except one, who gave birth 2,5 years ago.

### Data collection

Individual semi-structured interviews were conducted with the six Norwegian mothers in the timeframe September 2021-January 2022. Two of the interviews were conducted in person and four were conducted online (Microsoft Teams), of which three consented to have the camera on during the conversation. The interviews (50–90 min) were audio recorded and transcribed verbatim.

The first author conducted all the interviews. Topics discussed were how DRA is experienced and how the condition affects different aspects of day-to-day life. An interview guide was prepared beforehand with open-ended questions [24] based on the research questions (i) How do mothers with DRA experience everyday life? (ii) What does it mean to have DRA as a mother in relation to one’s own body? (iii) How does presence of DRA affect lived life regarding limitations and external influence? The mothers were asked to reflect on their own experiences, feelings, and thoughts and describe real life situations and examples. The interview guide has been included as a supplementary file.

### Analysis

The transcribed interviews were analyzed using systematic text condensation (STC) and an inductive approach was implemented [25]. STC is a thematic, cross-case method suitable for exploratory analysis of text data. The procedure is a four-step method. First, the interviews were read several times to grasp the main entity and identify preliminary themes. Preliminary themes were the *relationship to one’s own body, imperatives of everyday life, health conditions*, *social relationships* and *obtaining knowledge.* Second, meaning units regarding the research questions were identified and sorted according to established code groups. Third, meaning units of each code group were sorted into subgroups and condensates were abstracted from each subgroup. Table 1 provides examples of meaning units and codes in different themes and sub-groups. Fourth, the condensates were re-conceptualized generating a synthesized description of the lived experiences among mothers with DRA, structured in result-categories and sub-categories. These are presented in Table 2.

**Table 1:** Examples of meaning units and codes in different result-categories and sub-categories

Preliminary theme	Meaning unit	Code	Sub-categories	Result-categories
Relationship to own body	“I am trying to make my belly least possible visible, which is easy now during the winter when one is wearing a lot of clothes”	Wanting to hide belly	An ambivalent relationship to my body as a mother	*A changed belly – on self-image and social interactions*
Relationship to own body	“I have gained a lot of respect for my body. I have become very fond of it in all this.”	Finding solace in what body has managed to achieve	An ambivalent relationship to my body as a mother	*A changed belly – on self-image and social interactions*
Health conditions	“bending over to empty the dishwasher or tying my shoes… all that puts pressure on my belly is challenging. Then I can get nauseous and sick”	Restrictions in everyday activities	Physical limitations and tackling the demands of motherhood	*DRA – intertwined with health issues and physical limitations*
Imperatives of everyday life	“I have to sit down for a while, just try to relax a little, before I move on to the next chore. But I always do them [chores], because I know they have to be done! I don’t find other solutions!”	Prioritizing family and household regardless of own ailments	Physical limitations and tackling the demands of motherhood	*DRA – intertwined with health issues and physical limitations*
Social relationships	“Some influencers have made this more normal, which makes me not the only one walking around with it.”	Being part of a community - togetherness	The positives and negatives of social media	*The path to obtaining knowledge and understanding of DRA*
Obtaining knowledge	“Back then I didn’t understand what it was. But I was pregnant with my first, 4 years ago. I had gotten to about week 18 [when I first noticed the DRA bulge].”	Having no previous knowledge of DRA	From uncertainty to trustworthy knowledge	*The path to obtaining knowledge and understanding of DRA*

### Ethics

This study was approved by the Regional Committee for Medical Ethics in Norway (278,573). The mothers were provided with written and verbal information and signed a consent form prior to the interviews. To maintain confidentiality, audio files and transcriptions were stored securely on a password required remote desktop, SAFE, at University of Bergen, to which only the authors had access. Fictional names were given to the mothers to maintain anonymity. During the interviews, it was attempted to set an accepting environment so the mothers would feel safe sharing own experiences. The possibility of follow-up conversations was available as one of the authors has advanced training in counseling and sexual health education. None of the participating mothers needed this.

## Results

An overview of the result categories, with sub-categories is presented in Table 2. The categories are described and illustrated with quotations from the participants.

**Table 2:** Overview of the result-categories and sub-categories emerging in the analysis of the interviews

Result-categories	Sub-categories
The path to obtaining knowledge and understanding of DRA	From uncertainty to trustworthy knowledge A relief – yet a frustration The positives and negatives of social media
DRA - intertwined with health issues and physical limitations	DRA – woven into other health issues Physical limitations and tackling the demands of motherhood
A changed belly – on self-image & social interactions	An ambivalent relationship to one’s own body as a mother Other people’s opinions

### The path to obtain knowledge and understanding of DRA

The mothers struggled navigating in the information maze, causing uncertainties and frustration when attempting to learn more about DRA. Yet, it was reassuring once one had found a reliable source of information and had DRA diagnosed. Social media was associated with both negative and positive feelings. It had a crucial role in the information obtaining aspect as well as in how the belly was experienced by the mothers.

### From uncertainty to trustworthy knowledge

DRA was first noticed as a physical discomfort or a visually changed belly, which eventually lead all mothers reaching out to healthcare services to have it confirmed. Some mothers feared that the change was potentially a serious illness. Not having the typical symptoms listed online made the mothers uncertain whether they had DRA or not. The mothers struggled finding health care personnel with knowledge about DRA. Yet, once this was achieved there was a sense of reassurance, which made it easier to accept their bellies and shaping reasonable expectations. Having found a reliable source of information, the need of turning to other sources was reduced.

*Finally, I have someone I can trust. Someone who could say something about what I can do and give me realistic expectations as in what results I can expect from the exercises.* (Alice)

#### A relief – yet a frustration

Having DRA diagnosed was a relief, there was now a reason for the changed belly. Some had it diagnosed as a “coincidence” during a health examination, and several realized that they had had the condition since after their first pregnancy. Yet, the mothers said they had not received the help they had hoped for. Generally, it was perceived there was no treatment, the waiting time for surgery was endless and various remedies made their situation worse. Some experienced receiving contrasting information from different healthcare professionals. All this led to frustration. One mother said it felt like it was not viewed as a real problem in the healthcare services. A hope for greater awareness and a changed attitude towards DRA was expressed.

*You know what, it’s actually something we care about! Even though it could have been worse, but that applies to everything. It’s not like you shouldn’t care about things just because things could’ve been worse.* (Bianca)

#### The positives and negatives of social media

Several mothers used social media as a source of information for DRA. Two mothers specifically stated not using social media for this purpose. Some experienced gaining more knowledge on the condition through social medias than search engines. Reading about other peoples’ experiences was important. One mother told she had seen commercials on social media and that way suspected she had DRA. The mothers pointed out they had never heard of the condition prior to diagnosis. However, the mothers experienced difficulties knowing whether the information they had obtained was reliable. For some, social media was seen as an honest source, giving a sense of togetherness or being part of a community. Social media was recognized as having the potential to create transparency and shed light on the condition.

*It’s an information channel, where I’ve obtained a lot of good, but also a lot of bad information. Some influencers have made this more normal. That has probably made it easier for me to deal with, that I’m not the only one in the world that has it.* (Carol)

For others it was perceived as a superficial platform, where inaccurate information was spread. Due to scarce knowledge on DRA, some mentioned feeling fear of saying something inaccurate on social media when sharing own experiences.

*I feel that social media defeats its own purpose. As I live with it, I don’t really have the need to see other people struggling with the same.* (Darlene)

### DRA – intertwined with health issues and physical limitations

Having DRA in addition to other health issues complicated the situation. It was challenging knowing the origin of symptoms yet easy to ascribe new or old, but unexplained symptoms to DRA. Despite various physical limitations, the everyday imperatives of taking care of children and a household were almost always performed. Yet, the feeling of having lost valuable time with own children was distressing, as one now could not play in the same way and as much as previously.

#### DRA – woven into other health issues

DRA was intertwined with other health issues as all mothers in this study presented health issues of varying degree and a few had a chronic somatic disease or long-term health problem. A few mothers expressed having to deal with DRA as intolerable when other health issues had to be dealt with, yet for others DRA was the least of their problems. Having to cope with several health issues simultaneously was draining for some.

*There are enough strains in my life. I have chronic illnesses that I can’t do anything about. If I only had one less of these things… Maybe that is why I’ve raised my threshold for self-restraint, as I am already dealing with so many restrictions. I can’t let this hold me back even more, the little I can do.* (Bianca)

A few mothers had already decided on surgery. One mother explicitly told that the hardest part about having surgery was the concurrent decision to not have more children. Concerns of having surgery were the procedure itself, the healing process, scars, and having pain or being unable to carry things or do housework afterwards. Other concerns were the lack of social support network and uncertainty whether it would be covered financially by the public health system. The option of surgery was acknowledged; however, it was perceived as both terrifying and not desired if it could be avoided. In cases where mothers had health issues resolved yet still had persisting physical ailments, such ailments were ascribed to DRA.

*When I carried her in the car seat, it actually hurt so much I almost cried. The car seats are quite heavy, but I don’t recall them being that heavy. I had to take her out of the seat if I’d go to the store or the health center. I simply wasn’t strong enough getting the car seat out. I think that has a lot to do with DRA. Because then you use your upper body. It’s never been a problem before.* (Elisabeth)

#### Physical limitations and tackling the demands of motherhood

Several mothers restricted themselves in some activities and movements due to discomfort or pain, especially when pressure was applied on the belly. Having a body not capable of doing the same as previously was disappointing. The changed body had led to a decreased physical activity, which was perceived as a great loss. Despite explaining being restricted in some movements, and sometimes having to take extra breaks, the mothers perceived housework as a necessary chore. They were always completing the chores, either just getting on with it or finding other solutions. The same perspective was applied when taking care of their children. The children were always prioritized, even if it caused aches or pains. Yet, simultaneously some wished they had more time and energy to play with their children.

*I haven’t gone on mountain hikes since I got pregnant with my first. It’s a great loss, I miss being active […]. Crawling on the floor and through a tunnel is something I’ve always done when playing with kids before. I’ve always participated. It hurts to go through not being able to do the same as before. That has been painful because they are my children!* (Darlene)

### A changed belly – on self-image & social interactions

Having a changed belly affected the mothers’ self-image, which manifested in choice of clothing, repressive behavior, a tendency to social isolation, and feelings of shame and guilt. Some mothers felt vulnerable to other’s gaze. Despite experiencing physical limitations and health issues, being thankful for having a functioning body in everyday life and taking pride in its ability to bear a child was also expressed amongst the mothers.

#### An ambivalent relationship to one’s own body as a mother

The changed belly was perceived by several mothers as ugly and unflattering, which resulted in wearing clothes to hide the belly. The change in clothing was expressed by a mother as a loss of a part of her identity. She was one of the mothers who struggled more than the others in accepting their changed belly and some mentioned an attempt in ignoring their bellies.

*It’s something that is not as it should be. I can accept my body as it is, but this is a defect! One thing is being born [with a certain body type], another is when something is broken.* (Bianca)

Some mothers started realizing during the interviews that they had been downplaying many of their issues. Nevertheless, the mothers managed to find solace in their body’s achievement and felt pride in having brought forth children.

*At the same time, I’ve gained a lot of respect for my body. It’s a bit typical; you may not appreciate the things you´ve had before you lose some of it. It is kind of like that feeling, that I’ve in a way become very fond of [my body] but find it difficult, in a way, not noticing my belly when looking in the mirror.* (Carol)

A fear of contributing to their children developing a negative body-image was mentioned. Some mothers were very much aware of not being too much concerned by the look of their changed belly. Nevertheless, the mothers focused on how they could support their children in developing a healthy body-image.

*I hope my imperfect belly can be a perfect example for them.* (Alice)

#### Other people’s opinions

Some mothers had received hurtful comments. Yet, it was emphasized one did not want others to feel bad for asking so they rarely answered the comments. For some mothers DRA had contributed to a tendency of social isolation. In instances where social gatherings had been avoided some expressed, they needed their energy at home while a few stated they did not want others to see or comment on their belly.

*I often got comments and looks; “Are you pregnant?”. No, it’s just the gap between my abdominal muscles that haven’t closed yet, I went around saying. Then they would apologize. It’s fine [I said]. But deep down it really wasn’t!* […] *When the lockdown came, I remember thinking to myself: “Thank God, now I won’t have to deal with all the questions!”.* (Darlene)

Some mothers expressed feelings of embarrassment, especially when talking about the condition among friends, while others also felt guilt. They pondered whether they had brought this upon themselves or could have done something to prevent it. Several mothers felt that people mainly reacted to the aesthetic side of the condition. However, some mothers emphasized this was not a primary concern and therefore did not feel understood.

## Discussion

In this qualitative study mothers’ experiences living with DRA were explored. The empirical data indicates that DRA have an impact on everyday life, physically, emotionally, and socially.

### The path to obtaining knowledge and understanding of DRA

The mothers in this study experienced receiving contrasting information and perceived there was no treatment for DRA during their encounter with healthcare professionals. The mothers experienced not being listened to, taken seriously, or even felt dismissed. This finding is in line with a Swedish study, reporting that women, to a greater extent than men, experience being met with disrespectful behavior, like nonchalance, disbelief, feeling not listened to, or frequent interruption [20]. Women in the present study reported a sense of relief when they received a DRA diagnosis, despite the prolonged duration it took to obtain it. Previous research indicates that on average, women experience that it takes a long time to receive a correct diagnosis [26]. One of the reasons for this may be that when these women present their symptoms, they are not taken seriously. Moreover, the attitude and beliefs that women in general exaggerate their pain [27], may cause inadequate symptom management in health care. Due to a lack of evidence-based information or guidelines, not only are the mothers unable to obtain authorized information on DRA but healthcare professionals are unable to provide evidence-based care [14]. The uncertainty among healthcare professionals as to whether DRA is a clinical health problem or a psychosocial or functional problem, and how to treat DRA may lead to adopting attitudes easily perceived as patronizing by the mothers [14]. Achieving a certain level of knowledge and personal skills as well as confidence regarding health may determine a person’s motivation and action to take lifestyle changing steps to improve personal health [17]. By strengthening capacities and skills, individuals can through the empowerment process express needs, be involved in decision-making and act towards meeting those needs [17]. Since health literacy may improve people’s access and capacity to utilize health information, it is fundamental to empowerment [17, 18]. In line with the conceptual model of health literacy [18] providing easily accessible evidence-based knowledge to both healthcare professionals and mothers could contribute to better health outcomes and a more satisfactory provider-patient encounter.

The scarce knowledge about DRA was an issue among mothers. The mothers in the present study perceived it challenging to find a professional with knowledge of the condition. Yet, unique to this sample was that all mothers eventually had found such a healthcare professional. Emotionally accepting a disease can help a person cope with the struggles that arises with it and view the disease as it is, a distressing physical change and not a personal failure [28]. Attaining a trustworthy source of knowledge seemed to aid these mothers in emotionally accepting their condition. The changed belly was now easier to accept, and more reasonable expectations could be shaped. Studies find that health literacy is empowering, and in the present study knowledge seem to improve mothers’ access and capacity to utilize health information [18]. In accordance with the conceptual model of health literacy [18], an established trustworthy source supported the mothers in proceeding the process of accessing, understanding, processing and applying health information on DRA.

Sourcing reliable information about DRA was in general a challenge for the mothers in this study. This has also been shown in a previous study among women with DRA [16]. In line with studies of postpartum women [29, 30], the mothers in the current study expressed how social media was a superficial platform making them compare themselves to others, which influenced their self-image. This can further be linked to findings that social media also may give rise to fear, distress and feelings of not fitting in [31]. However, the mothers in the current study also connected with other women with DRA through social medias, which lead to a sense of belonging. These increased feelings of belonging and connection through social media has been found in previous studies among young adults living with illnesses or chronic conditions [31]. Considering that some mothers in the current study felt they gained more knowledge on social media than through search engines, it was paradoxical that almost all were aware that inaccurate information easily spread on social media. Social media is a potential tool to spread health information to mothers, but should be cautiously approached as the quality of the information provided may also affect the dimensions of health literacy [18]. However, social media may contribute to provide evidence-based guidelines and encourage postpartum women to follow such guidelines. Producing and distributing reliable, evidence-based knowledge on the condition, to further support outlining future guidelines, should therefore be prioritized.

### DRA – intertwined with health issues and physical limitations

In line with findings that experiences of illness makes people vulnerable to a greater extent and may even alter one’s self-conception [32], we found an increased feeling of vulnerability among the mothers in the present study. Interestingly, even though some struggled with the aesthetic side of the condition, several mothers in the present study emphasized that this was not their primary concern. Having DRA either reinforced the perceived effects of other health issues, or the perceived effect of DRA was reinforced when having other health issues. The variations in how DRA and simultaneous health issues were perceived by the mothers, could be due to individual resilience [33]. Resilience is a protective process that includes the coping mechanisms of an individual and the ability to resist the impact of negative events and/or factors while maintaining normal function [33]. Being unsure if DRA could be treated, and not knowing what to expect regarding improvement of the condition, may also have influenced the mothers’ thoughts about DRA’s link to other health issues. To fully understand the association between DRA and other health issues, there is a need for further studies that investigate the interaction between possible implications of DRA and co-current health issues.

Among the mothers in the present study there was uncertainty of the reason behind their changed belly. Along with scarce knowledge on DRA, this uncertainty may lead to construction of explanatory models [34] as some mothers presented here. Explanatory models are mediated by cultural and social contexts and can lead to a conflict between the patient and provider, as the condition may be perceived contradictory, causing them to talk past each other [34]. This is probably another reason for the perceived negative healthcare encounters among the mothers in the current study. Nevertheless, DRA is a multifaceted condition affecting many aspects of day-to-day life, like physical, emotional, and social, which is consistent with the findings of Eriksson Crommert et al. [16].

Evidence show that when DRA causes great functional or cosmetic disability, surgery may be an option, which is considered safe and effective [35]. Nevertheless, it appears to be a lack of consensus concerning the indications for surgery [35]. In the current study, several mothers considered surgery, which brought forth concerns regarding economy and social support. Norway has universal health coverage, in which not all cosmetic surgery is covered. Investigating the need for and clarifying the indications for surgery may supply clear guidelines for when a DRA surgery is eligible for health coverage. Furthermore, such information may support healthcare professionals in providing information that aids the mothers in applying relevant information to health, which further strengthens their health literacy and empowerment [17, 18].

### A changed belly – on self-image & social interactions

The mothers in the present study reported body dissatisfaction and aesthetic discomfort due to DRA. However, this is not unique to the experience of DRA, but seem to be quite common among postpartum women, which may be influenced by socio-cultural ideals and social media [2, 29, 30]. Furthermore, it is suggested that body dissatisfaction may be more prevalent among women with DRA than those without [16]. Even so, the mothers in the current study expressed pride and gratefulness in their bodies’ achievements (a healthy child had grown in one’s own body), and also that appearance was less important than having a functioning body. Similar findings are presented previously in postpartum women [30]. Some mothers perceived their changed bellies as ugly and even defect, yet others found it easier to accept, which also could be explained by resilience. By focusing on personal strengths and qualities mothers may be aided in their ability to cope with a stressful situation, in this case the type of stress body image dissatisfaction could impose [33].

The mothers in this study reported feelings of guilt and shame. The size and/or severity of DRA, other health issues and hurtful comments could explain the variations in tolerating living with DRA. Some mothers realized they had downplayed some of their issues in their mind, which was interpreted as a form of repression, perhaps due to the feelings of guilt and shame, which also could further explain parts of the social isolating tendency among some of the mothers. Moreover, caring for infants and toddlers poses its own challenges in addition to possibly affecting social life.

Guilt and shame due to a condition may also cause disruption in mothering [36]. Being unable to play with their own children as they wished, in addition to the change in activity and function, were perceived as losses by the mothers. The children’s needs always came first, which is consistent with the findings that women who have been disrupted in their mothering due to illness, firstly view themselves as mothers and secondly as patients [36]. Considering the persistent feeling of not being understood among the mothers and effect of DRA on overall wellbeing, generating a greater understanding of the condition may remove some of the taboo some of the mothers experienced.

### Methodological considerations

To increase internal validity, member checking during the interviews was performed. Follow up questions like: “What do you mean by…?” or “Do I understand correctly that…?” were asked to validate the interpretation of the mothers’ experiences of and reflections on living with DRA [24]. Furthermore, short summaries at the end of the interviews gave the mothers room to clarify, correct, and provide additional information. To further enhance validity, transcripts and text-close interpretations could have been shared with the mothers [37]. This was not done due to time constraints, being a part of a master’s thesis.

Reflection on information power could have been more systematically conducted [23]. A sample size of six participants may be considered a limitation in this study. According to Malterud et al. [23], if the purpose is broad, a qualitative study may benefit of having a larger sample size. In our study, the aim was limited to exploring some mothers’ experiences living with DRA, not covering the whole spectrum of possible experiences of this phenomenon. However, variation in some important background factors, and aiming for rich descriptions in the single interview, resulted in data material with varied experiences on core themes, and rich on examples. The participants also held qualities that were considered specific to the aim of the study, thus strengthening the information power. Still, we will not rule out that more information may increase saturation. However, the conceptual model of health literacy, together with the analysis enabled a more extensive interpretation of the data [23].

Recruitment from a sample from another study could be considered narrowing the recruitment base and therefore a limitation. Yet, mothers who themselves contacted the project were later recruited, which proved to provide this study with a greater variation in background factors. Mainly in greater variation of IRD but also in other characteristics, like a broader age span, and living in two different regions of Norway. A greater diversity among participant is considered to strengthen the information power [23]. It is likely that participants that recruited themselves were concerned about their DRA, and wanted to know what increased and decreased the gap, and/or experiencing DRA related restrictions and health issues. The participants in this study expressed a wide variety in concerns about their DRA.

Transferability of findings and insights from this study relate to pragmatic validity [24]; findings are deemed relevant by mothers with DRA in similar contexts, and of health care providers who try to advice and help. Given our society is globalized and connected through social media and a great focus on appearances [29, 30], some findings in this study may apply to mothers with DRA in a western context.

## Conclusion & clinical implications

DRA is a multifaceted condition affecting many aspects of day-to-day life, like physical, emotional, and social. The mothers in this study found it challenging to acquire evidence-based information regarding the condition. This caused uncertainties, prompting mothers to seek, not necessarily evidence-based, information through social media channels. In this study, DRA showed to co-occur with various physical limitations and health issues, making it difficult to know whether symptoms were due to DRA or not. A great concern for the mothers in this study was the affected self-image, yet an ambivalence towards one’s own body as a mother.

A holistic and person-centered approach should be strived at in encounters with mothers with DRA in a healthcare setting, as this could narrow the contradictions in the perceived need and care between provider and user/mother.

Contributing to a wider understanding of DRA, the results from this study might aid healthcare professionals in exploring concerns, providing support and evidence-based information to these mothers. Furthermore, studies identifying women at risk of developing DRA may enable healthcare professionals to implement targeted interventions and strategies to high-risk individuals. By understanding specific factors associated with DRA in women, more effective preventive measures can be offered, which ultimately may improve these women’s health outcomes and quality of life.

### Electronic supplementary material

Below is the link to the electronic supplementary material.

Supplementary Material 1

## Data Availability

Data generated consists of quotes by the interview subjects that contain information, which could reveal the identity of the subject. The datasets used in the current study are therefore not publicly available.

## Abbreviations

DRA: diastasis recti abdominis
IRD: inter recti distance

## References

[1] Fernandes da Mota PG, Pascoal AG, Carita AI, Bø K (2015). Prevalence and risk factors of diastasis recti abdominis from late pregnancy to 6 months postpartum, and relationship with lumbo-pelvic pain. Man Ther.

[2] Cardaillac C, Vieillefosse S, Oppenheimer A, Joueidi Y, Thubert T, Deffieux X (2020). Diastasis of the rectus abdominis muscles in postpartum: concordance of patient and clinician evaluations, prevalence, associated pelvic floor symptoms and quality of life. Eur J Obstet Gynecol Reprod Biol.

[3] Sperstad JB, Tennfjord MK, Hilde G, Ellström-Engh M, Bø K (2016). Diastasis recti abdominis during pregnancy and 12 months after childbirth: prevalence, risk factors and report of lumbopelvic pain. Br J Sports Med.

[4] Fuentes Aparicio L, Rejano-Campo M, Donnelly GM, Vicente-Campos V (2021). Self-reported symptoms in women with diastasis rectus abdominis: a systematic review. J Gynecol Obstet Hum Reprod.

[5] Cavalli M, Aiolfi A, Bruni PG, Manfredini L, Lombardo F, Bonfanti MT (2021). Prevalence and risk factors for diastasis recti abdominis: a review and proposal of a new anatomical variation. Hernia.

[6] Beer GM, Schuster A, Seifert B, Manestar M, Mihic-Probst D, Weber SA (2009). The normal width of the linea Alba in nulliparous women. Clin Anat.

[7] Parker MA, Millar LA, Dugan SA. Diastasis Rectus Abdominis and Lumbo-Pelvic Pain and Dysfunction-Are they related? J Women’s Health Phys Therapy. 2009;33(2).

[8] Benjamin DR, Frawley HC, Shields N, van de Water ATM, Taylor NF (2019). Relationship between diastasis of the rectus abdominis muscle (DRAM) and musculoskeletal dysfunctions, pain and quality of life: a systematic review. Physiotherapy.

[9] Spitznagle TM, Leong FC, Van Dillen LR (2007). Prevalence of diastasis recti abdominis in a urogynecological patient population. Int Urogynecol J Pelvic Floor Dysfunct.

[10] Fei H, Liu Y, Li M, He J, Liu L, Li J (2021). The relationship of severity in diastasis recti abdominis and pelvic floor dysfunction: a retrospective cohort study. BMC Womens Health.

[11] Gluppe S, Ellström Engh M, Kari B (2021). Women with diastasis recti abdominis might have weaker abdominal muscles and more abdominal pain, but no higher prevalence of pelvic floor disorders, low back and pelvic girdle pain than women without diastasis recti abdominis. Physiotherapy.

[12] Gluppe S, Ellström Engh M, Bø K (2022). Primiparous women’s knowledge of diastasis recti abdominis, concerns about abdominal appearance, treatments, and perceived abdominal muscle strength 6–8 months postpartum. A cross sectional comparison study. BMC Womens Health.

[13] Carlstedt A, Bringman S, Egberth M, Emanuelsson P, Olsson A, Petersson U (2021). Management of diastasis of the rectus abdominis muscles: recommendations for Swedish national guidelines. Scand J Surg.

[14] Gustavsson C, Eriksson-Crommert M (2020). Physiotherapists’ and midwives’ views of increased inter recti abdominis distance and its management in women after childbirth. BMC Womens Health.

[15] Benjamin DR, van de Water AT, Peiris CL (2014). Effects of exercise on diastasis of the rectus abdominis muscle in the antenatal and postnatal periods: a systematic review. Physiotherapy.

[16] Eriksson Crommert M, Petrov Fieril K, Gustavsson C (2020). Women’s experiences of living with increased inter-recti distance after childbirth: an interview study. BMC Womens Health.

[17] WHO. Health promotion glossary. Geneva; 1998. Report No. WHO/HPR/HEP/98.1

[18] Sørensen K, Van den Broucke S, Fullam J, Doyle G, Pelikan J, Slonska Z (2012). Health literacy and public health: a systematic review and integration of definitions and models. BMC Public Health.

[19] Haile VT. Gender as a factor in the Physician and Patient Interaction: from the Service Quality Perspective. J Mark Consumer Behav Emerg Markets. 2018.

[20] Upmark M, Borg K, Alexanderson K (2007). Gender differences in experiencing negative encounters with healthcare: a study of long-term sickness absentees. Scand J Public Health.

[21] Mota P, Pascoal AG, Carita AI, Bø K (2018). Normal width of the inter-recti distance in pregnant and postpartum primiparous women. Musculoskelet Sci Pract.

[22] Quinn PM (2015). Qualitative Research & Evaluation Methods: integrating theory and practice.

[23] Malterud K, Siersma V, Guassora AD. Information power: sample content and size in qualitative studies. In: Camic PM, editor. Qualitative research in psychology: expanding perspectives in methodology and design. American Psychological Association; 2021. pp. 67–81.

[24] Steinar K, Svend B (2009). Interviews - learning the craft of qualitative research interviewing.

[25] Malterud K (2012). Systematic text condensation: a strategy for qualitative analysis. Scand J Public Health.

[26] Westergaard D, Moseley P, Sørup FKH, Baldi P, Brunak S (2019). Population-wide analysis of differences in disease progression patterns in men and women. Nat Commun.

[27] Samulowitz A, Gremyr I, Eriksson E, Hensing G (2018). Brave men and emotional women: a theory-guided literature review on gender Bias in Health Care and gendered norms towards patients with Chronic Pain. Pain Res Manage.

[28] Büssing A, Matthiessen PF, Mundle G (2008). Emotional and rational disease acceptance in patients with depression and alcohol addiction. Health Qual Life Outcomes.

[29] Nagl M, Jepsen L, Linde K, Kersting A (2021). Social media use and postpartum body image dissatisfaction: the role of appearance-related social comparisons and thin-ideal internalization. Midwifery.

[30] Ingrid L, Målfrid R. «Ikke for liten. Ikke for stor» – unge postgravide kvinners erfaringer gjennom kroppslige endringer. Fysioterapeuten. 2020(8):76–81.

[31] Wilson C, Stock J (2021). Social media comes with good and bad sides, doesn’t it?’ A balancing act of the benefits and risks of social media use by young adults with long-term conditions. Health (London).

[32] Bluhm R (2012). Vulnerability, health, and illness. Int J Feminist Approaches Bioeth.

[33] Babić R, Babić M, Rastović P, Ćurlin M, Šimić J, Mandić K (2020). Resilience in Health and Illness. Psychiatr Danub.

[34] Kleinman A, Eisenberg L, Good B (1978). Culture, illness, and Care: clinical lessons from Anthropologic and Cross-cultural Research. Ann Intern Med.

[35] ElHawary H, Abdelhamid K, Meng F, Janis JE (2020). A Comprehensive, evidence-based literature review of the Surgical treatment of Rectus Diastasis. Plast Reconstr Surg.

[36] Vallido T, Wilkes L, Carter B, Jackson D (2010). Mothering disrupted by illness: a narrative synthesis of qualitative research. J Adv Nurs.

[37] Malterud K (2001). Qualitative research: standards, challenges, and guidelines. Lancet.

