# Supplementary material for: Mothers’ experiences living with diastasis recti abdominis – an interview study
Source: BMC Womens Health. 2024 May 17;24:292. doi: 10.1186/s12905-024-03131-x (PMC11100213; doi:10.1186/s12905-024-03131-x)
Supplement: Supplementary file 1 — Supplementary Material 1 [file 12905_2024_3131_MOESM1_ESM.docx]

**Date, time and place**:

*Welcome the interviewee. Introduce myself and the project, explaining my task and role as an interviewer. Emphasize that I'm seeking detailed descriptions of real-life examples and situations. Clarify that participants can withdraw from the study at any time. Ask if there are any questions.*

*Start recording.*

**Background information**, such as: age, household/number of children, approximate time since last childbirth, IRD.

**Everyday life** *(How do mothers with DRA present experience everyday life?)*

- Can you recall the first time you noticed, felt, or became aware of having DRA? What were your initial thoughts and feelings?
- Describe what a typical day looks like for you. In which situations do you become aware of DRA, and when do you completely forget about it?

**Relationship to own body** *(What does it mean to have DRA as a mother in regard to* *own body?)*

- How has DRA affected you in how you view your own body?
- Self-image, body-image and confidence
- Have you experienced any expectations related to DRA? If so, can you tell me more about that?

**Limitations and external influences** *(How does the presence of DRA affect lived life in regard to limitations and external influence?)*

- How has DRA impacted your physical activity habits?
- How has social media influenced your view on DRA?
- Has DRA led to changes in social interactions? If so, in what ways and can you provide an example?
- Relationships and intimacy
- Has DRA had an impact on your role as a mother? If so, how?

*Ask if there is anything the participant wants to add. Stop recording. Thank participants for participating and assure confidentiality, explain data storage and who has access to it. Also give information about potential future interviews*
